# Expert Testimony in the Hayden Trial

**Published:** 1880-02

**Authors:** Wm. T. Belfield

**Affiliations:** Demonstrator of Physiology, Rush Medical College, Chicago


### ARTICLE IV.

EXPERT TESTIMONY IN THE HAYDEN TRIAL. By WM. T. BELFIELD, M.D., Demonstrator of Physiology, Rush Medical College, Chicago.

On the afternoon of September 3, 1878, the dead body of Mary Stannard was found a short distance from her father's house, near Middletown, Conn. The county coroner was notified, an inquest held, and a post-mortem examination made. The latter revealed a large bruise on the side of the head, and a deep incised wound of the throat, whereby the common carotid artery and internal jugular vein of one side had been divided; also, a severe contusion of the left hand. There was found a small pool of blood beside the body. The examination, made by local physicians, appears to have been quite superficial and incomplete, eliciting no further information of value. Speculation, as to the perpetrator of the crime, was rife, some even including the theory of suicide. Various facts, however, directed suspicion toward a Rev. Mr. Hayden, a near neighbor, in whose family Mary had been employed as a domestic. It was more than hinted that Hayden had murdered the girl in order to prevent the natural consequences of a criminal intimacy with her; and it was suddenly noised abroad that Hayden had, on the day of the murder, purchased an ounce of arsenic of a Middletown druggist, ostensibly to poison rats.

A second examination of the remains was made eight days after death, conducted this time by men of established reputation. The uterus gave evidence of disease, but not of impregnation; the right ovary presented a tumor as large as a walnut, which was reserved for microscopical examination; the stomach, which exhibited the appearances of acute inflammation, and several other organs, were removed and sent to a chemist, who found 90 grains of undissolved arsenic in the stomach, and traces of the drug in the other organs. Besides this account of the examination, the prosecution has proven the purchase of the arsenic, by Hayden, just previous to the murder; the girl's avowal to certain female friends of her pregnancy, and that, too, by Hayden; her statement that she had conversed with Hayden as to the best means for avoiding discovery; that Hayden had promised to procure for her some "quick medicine;" that she was going to meet him that afternoon to get this medicine; and finally, it is proven that she took with her a napkin and safety-pin, both articles having been found, indeed, on her dead body.

The theory of the prosecution is simply that she met Hayden as agreed, received from him a dose of arsenic, and was subsequently stabbed with his pocket-knife. The defense offer no theory of the murder, but content themselves with presenting objections to the evidence against the prisoner.

My purpose is to discuss the questions of expert testimony in their scientific aspect only, without regard to their legal bearing upon this trial.

*First.*—Is it possible to identify a given specimen of arsenic? Dr. E. S. Dana says, yes, within certain limits—a statement indorsed by Prof. Wormley, of Philadelphia. Dr. Dana affirms that the varying temperature at which the arsenic vapor is condensed, in the process of manufacture, and the varying fineness of the subsequent grinding, cause such diversity in the size of the crystals and in the proportion of fragments, as to render identification easy—basing his conclusions chiefly on three months' residence at the arsenic factories of England, and including the examination of one hundred and ten specimens.

This assertion, apparently so captivating and satisfactory, must be dismissed as "not proven." The arguments against it are several: the lack of experience in the examiner; the admission, by Dr. Dana himself, that several specimens may be subjected to similar conditions in the process of manufacture, and may hence be undistinguishable; the fact that the same jar may contain portions of different packages; the plausible assertion of practical millers, that the particles are often of variable fineness, even at a single grinding, in consequence of a lack of parallelism between the stones, and of irregularities in their surfaces. These facts force us to the conclusion that while there are wide differences in the microscopical appearances of different specimens of arsenic which render *distinction* sometimes possible, yet *identification* is, as yet, impossible.

*Second.*—Is it possible to determine by the microscope that a given stain does or does not consist of human blood? On this point were obtained the opinions of two leading experts—Surgeon Woodward and Dr. Treadwell. It is a matter for congratulation and pride to microscopists, that these two gentlemen, representing, as they are reputed to do, the extremes of conservative and progressive opinion in this matter, should differ in no essential point as to the technicalities involved. The differences of opinion, important as they are, arise from the greater experience, and hence, very naturally, the greater caution of Col. Woodward. They agree that while the fresh blood of man can be generally distinguished from that of most of the domestic animals, and from that of all animals not mammals, yet there are certain animals whose blood-corpuscles are so nearly identical in size with those of man as to render positive distinction by the microscope impossible. Dr. Woodward, however, after alluding to the extreme variability in size of the corpuscles, in the same as well as in different individuals, objects to the assumption of $\frac{1}{3200}$ inch, or any other figure, as the exact average of human corpuscles. He insists upon the impossibility of knowing the actual average, since successive examinations give varying measurements. In consequence of this fact, and of the further impossibility of assigning limits to the possible size of individual corpuscles, he urges the necessity of extreme caution; and insists that when attempting to distinguish corpuscles whose asserted "averages" (usually Gulliver's) differ but little—those of man and the dog, for example—the microscopist shall speak in terms of probability only. These remarks apply to blood freshly drawn upon glass slides; the uncertainties are much increased if the corpuscles to be measured have been obtained from a stain upon cloth, a clot, etc., and have been artificially "restored" to their former size. In this case the chances of success are diminished by the introduction of a new and important source of inaccuracy.

These sentiments will, I think, meet the approval of every conscientious microscopist, in part at least even of Dr. Treadwell himself. For I have in my possession a letter from Dr. Treadwell in which he expressly disclaims the ability either to identify dried human blood or to distinguish human from canine blood. Dr. Richardson, the well-known Philadelphia expert, makes the same statement for himself.

Dr. Woodward then proceeds to criticise Dr. Treadwell's assurance in basing an opinion upon an average of 15 corpuscles (found in the thumb-niche of Hayden's knife-blade) as entirely unwarranted. Dr. Treadwell maintains that the microscopist needs only 15 or 20 corpuscles in order to ascertain a fair average, and that he can "restore" dried corpuscles to their exact size. He then assumes the aggressive and criticizes Dr. Woodward's trust in the measurement of the photographic pictures of blood-corpuscles instead of the images as seen in the microscope.

This testimony is most opportune. In these days, when the laxity of the bench and the unscrupulous eagerness of the bar put a premium upon reckless extravagance of assertion and even falsehood on the part of expert witnesses; when a man is permitted to swear that he can not only distinguish human blood, but can even identify an individual by the size of the blood-corpuscles; in these days it is especially fortunate that we receive from the foremost microscopist in America—an unbiased witness—the exact state of microscopical science in this matter. It is to be hoped that the medical profession will do its part—a considerable one—in the propagation of the truth, and that there will result a more general knowledge of what the microscope can *not*, as well as what it *can* do, in the detection of crime. In this connection it is interesting to note, though hardly pertinent to the trial, the assertion of both Drs. Woodward and Treadwell, that it is impossible, in the present state of science, to diagnosticate disease by any abnormalities of appearance in the individual corpuscles. (Let me remind the medical reader that this is not a denial of ability to recognize leucocythæmia, remittent fever, etc., which are distinguished, not by the peculiarities of individual corpuscles, but by their relative numbers and the presence of spirillum). This is, however, rather damaging to the claims of certain gentlemen that in consumption, syphilis, and perhaps other diseases, the corpuscles present characteristic appearances.

*Third.*—Can an ordinary ovarian cyst, no larger than a walnut, occasion the symptoms of early pregnancy? Strangely enough, two Yale professors testified in the affirmative, although, as was subsequently proven, there is no authority nor recorded instance warranting such an assertion. Every medical man (except the two professors) knows that ovarian tumors usually attain a larger size before attracting the patient's attention to their presence and *then* not by causing symptoms of pregnancy, but by their pressure on other organs and by the local pressure of the abdominal walls. It is by no means seldom that one finds, post-mortem, ovarian cysts larger than the one in question, whose existence had never been suspected by the patient, nor even by her medical attendant.

*Fourth.*—How much blood would flow from an incised wound in the common carotid artery of a woman of average weight? And how much would be required to saturate firm earth to a depth of two inches, and over a circular area eight inches in diameter? The answers to these questions were, of course, speculative, indefinite and worthless.

Several other questions were discussed in the testimony which are unworthy of attention from a scientific standpoint, however well they may have served the purposes of the attorneys in browbeating witnesses, and confusing jurors. Such were these: Could arsenic, inserted into the stomach of a corpse, so permeate the tissues by osmotic action as to occur in appreciable quantity in the brain and other organs? Would arsenic, placed in the stomach after death, induce the reddening and other signs of inflammation which it causes in the living stomach?

Now, while the expert testimony adduced will probably have but little effect on the verdict in this trial, it will have a far-reaching influence in determining the medical jurisprudence of the coming decade. For, besides the authoritative expression of opinion on several important points, we have acquired a clearer appreciation of several serious defects in our present system of expert testimony—defects which contain a large element of peril to society in consequence of the very importance of the expert witness. For the expert on the witness-stand is sometimes a far more potent figure than either judge or jury, and reads the sentence which the latter merely execute; hence the over-confident expert, even though of honest intentions, may be a more dangerous man to the community than the very criminal whom he prosecutes; while the dishonest expert is far more dangerous. I will enumerate a few of the evils which detract from the value of expert testimony as at present conducted:

*First.*—The employment of incompetent persons. This has received illustration not simply in the testimony with regard to the ovarian tumor, already referred to, but also in the preliminary trial, during which a *soi-disant* expert declared that a stain on a certain stone was made by blood. A subsequent examination by a competent microscopist demonstrated to the satisfaction even of the "expert" himself, that the supposed blood-corpuscles were the spores of a minute plant. Another witness at the preliminary trial denied the possibility of distinguishing human blood from that of a chicken.

*Second.* The employment of pretenders and charlatans.

*Third.* The partisan character of the testimony—the expert, like the lawyer, being hired by one party and being expected to testify first for the client, and second to the truth; the amount of the fee being in some cases measured by the expert's devotion to the cause and even dependent upon the result of the trial. Another unfortunate feature is the too frequent practice of introducing as evidence chemical experiments, microscopical exhibitions and the like, requiring special training and skill for their appreciation. To the juror of even average intelligence such a proceeding must savor largely of quackery, and must seem an attempt to manufacture reputation and authority by a cheap display of knowledge. The expert's testimony is procured because of his supposed familiarity with subjects of which the jurors are ignorant; and the weight carried by his evidence is dependent upon and largely proportionate to his skill and experience. In so far, therefore, as he attempts to fortify his evidence on some occult point by an appeal for corroboration to the jury, in just so far does he belittle himself and his opinion. True, it may occasionally happen that the expert can render intelligible to the jury the facts and laws upon which his opinion is based; yet the real object of the average scientific display in the court-room is not to enlighten jurors upon a question at issue but rather to impress upon them a due sense of the expert's importance.

In consequence of such influences as these it has come to pass that the variability and contradictory nature of medical evidence is a mere byword in the mouths of attorneys. That is, indeed, a desperate case in which the assistance of a medical witness can not be secured, while the standard of chemical, microscopical and other expert testimony is far from satisfactory.

It might be presumed that the dishonest or incompetent expert would be detected by opposing experts, and so he is ultimately; yet that does not necessarily destroy his business. Many a quack doctor who claims to cure cancer, consumption or drunkenness, does a thriving business because conscientious, reputable physicians admit their utter inability to perform such miracles. And so the quack microscopist who claims the ability to identify human blood, or an individual's hair, or to do anything else which conscientious, reputable microscopists admit is impossible, is largely patronized by States' attorneys and others who desire to establish just such points. A heavy responsibility rests upon the legal profession in this matter; for attorneys do not hesitate to employ for the accomplishment of their ends witnesses, expert and other, of whose moral worthlessness they are convinced, and in whose statements they have themselves no confidence. An instance of this kind came to my ears by the personal confession of the attorney, a well-known lawyer of this city, and I have no doubt that he could have lengthened the chapter, had he desired.

It is eminently desirable that these abuses shall be corrected; yet to propose a remedy would be premature until the popular realization of the evil calls for it. Perhaps we shall ultimately find the best way to be that adopted in Germany, where scientific experts are government officers whose comfortable salaries, paid for life or during good behavior, remove the temptation to evil or rather transform it into an impulse for good.

